# Species distribution and seasonal dynamics of equine tick infestation in two Mediterranean climate niches in Israel

**DOI:** 10.1186/s13071-018-3093-0

**Published:** 2018-10-16

**Authors:** Sharon Tirosh-Levy, Yuval Gottlieb, Dmitry A. Apanaskevich, Kosta Y. Mumcuoglu, Amir Steinman

**Affiliations:** 1 Koret School of Veterinary Medicine, The Robert H. Smith Faculty of Agriculture, Food and Environment, The Hebrew University of Jerusalem, Rehovot, Israel; 2 United States National Tick Collection, James H. Oliver, Jr., Institute of Coastal Plain Sciences, Georgia Southern University, Statesboro, Georgia USA; 3 Parasitology Unit, Department of Microbiology and Molecular Genetic, The Kuvin Centre for the Study of Infectious and Tropical Diseases, Hebrew University-Hadassah Medical School, Jerusalem, Israel

**Keywords:** *Hyalomma*, *Rhipicephalus*, *Haemaphysalis*, Horse, Epidemiology, Israel

## Abstract

**Background:**

Ticks are important ectoparasites of horses that can affect animal welfare and vector several infectious, including zoonotic, diseases. In order to investigate the species distribution, epidemiology and seasonal dynamics of ticks infesting horses in Israel, 3267 ticks were collected from 396 horses in 24 farms across the country from July 2014 to June 2015.

**Results:**

Ticks were found on 50% of the farms and on 25% of the horses, with *Hyalomma* being the most prevalent genus (70% of ticks). Pasture was the most prominent risk factor for tick infestation (99% of ticks, *P* < 0.001), and is represented here by two areas with a Mediterranean climate that differ in their environmental characteristics: the Golan Heights (GH, 74% of ticks); and the Carmel mountain ridge (CMR, 24%). Although these two sites are less than 100 km apart, the composition of the tick populations infesting horses differed significantly between them. In GH the most abundant tick species was *Hyalomma excavatum* (*P* < 0.001), while in CMR it was *Hyalomma marginatum* (*P* < 0.001)*.* The GH also hosted a more diverse tick fauna than the CMR, including *Haemaphysalis parva* (peaking in the autumn, *P* < 0.001) and *Rhipicephalus turanicus* (peaking in the spring, *P* < 0.001), which were not found at the other sites. A few *Rhipicephalus bursa*, *Hyalomma rufipes* and *Hyalomma turanicum* were also found on horses.

**Conclusions:**

The current findings can be used in epidemiological studies assessing the risk of tick-borne equine diseases in the area. Further analysis is needed to determine the specific distribution and habitat preferences of each tick species.

**Electronic supplementary material:**

The online version of this article (10.1186/s13071-018-3093-0) contains supplementary material, which is available to authorized users.

## Background

Ticks are important ectoparasites of horses that can affect animal welfare and vector several infectious, including zoonotic, diseases. In recent years there has been a growing interest in assessing the climate niches of different tick species in order to better understand the relationship between pathogens, vectors and hosts [1]. The survival of different life stages of ticks, as well as their successful reproduction, is dependent on a combination of environmental and biotic factors, including climate, vegetation and host availability. Some ticks also exhibit ecological plasticity and may easily adapt to changing climate and new habitats [2, 3]. Climate changes in recent years have led to changes in the distribution of several tick species and introduction of certain tick species and infectious agents into previously unaffected areas [2, 4–6].

**Fig. 1:**
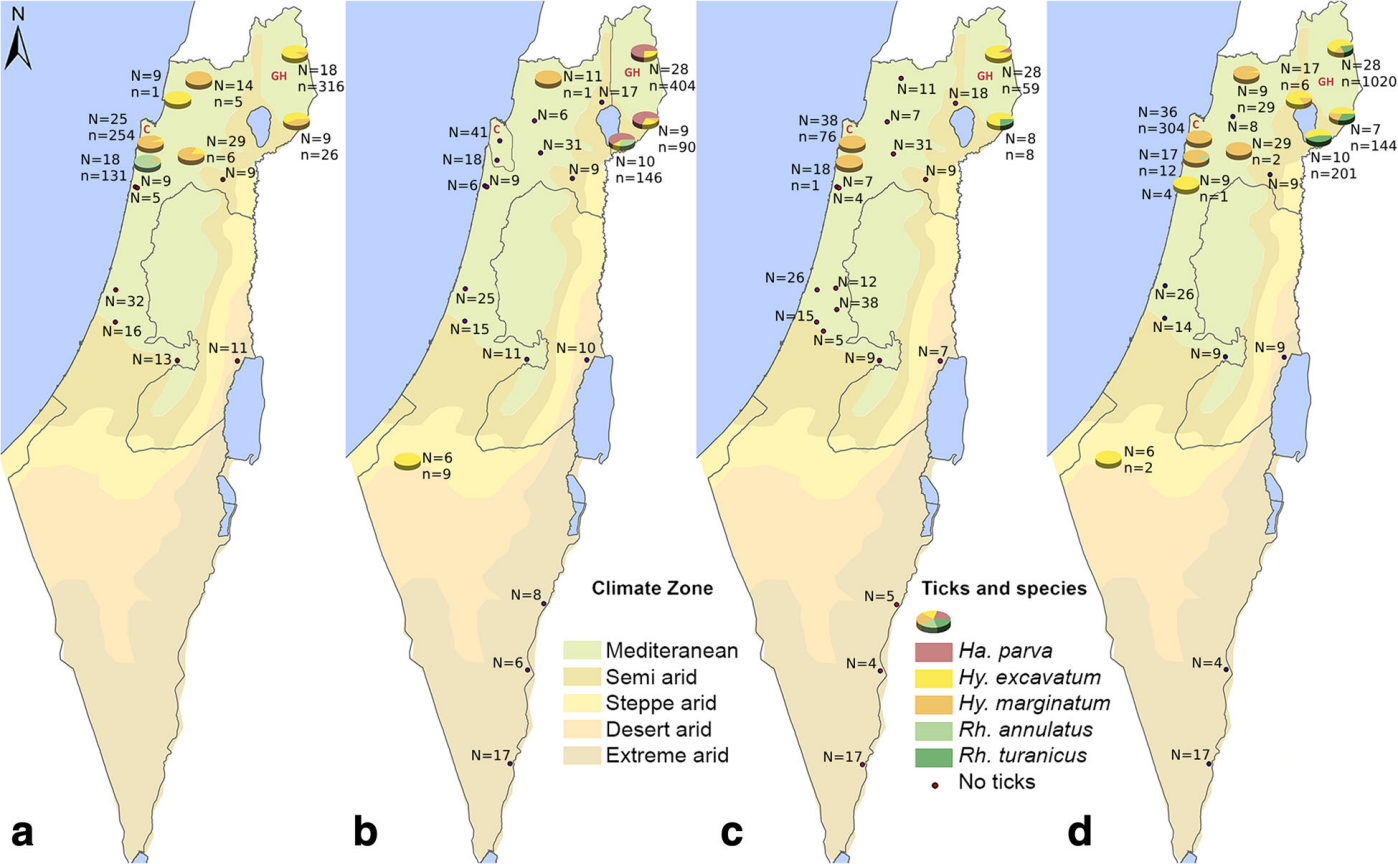
Geographical and seasonal distribution of equine ticks in Israel. Tick species distribution in each farm is depicted in a pie chart. **a** Summer (July-August 2014). **b** Autumn (November-December 2014). **c** Winter (February-March 2015). **d** Spring (May-June 2015). *Abbreviations*: N, number of horses scanned; n, number of ticks collected, C- Carmel mountain ridge, CG- Golan Heights

Israel is situated in the Mediterranean region, with a climate ranging from Mediterranean to extreme arid (Fig. 1) and provides a habitat for a variety of ixodid tick species. As a result, Israel has many endemic tick-borne diseases that affect humans and other animals, including pets and livestock (www.gideononline.com/ebooks/country/infectious-diseases-of-israel/). Equine piroplasmosis (EP) is highly prevalent in Israel [7–9], and zoonotic diseases such as Crimean Congo hemorrhagic fever (CCHF) and Mediterranean spotted fever (MSF) have been reported in the country and can be transmitted from ticks to horses [10–13]. Several tick species have been reported to infest horses in Israel. The ticks are mainly of the genus *Hyalomma*, including several possible vectors of EP and CCHF. However, none of the previous studies focused on horses as a host, and all were performed more than four decades ago [14–16].

The present study was designed as a large-scale survey of the horse population in Israel in order to describe equine tick infestation, identify risk factors, and explore the environmental and climatic factors associated with different tick species.

## Methods

### Tick collection and identification

Ticks were collected from horses at 24 farms selected to represent different geographical and climatic areas in Israel. Collections were performed every three months to represent all seasons: summer, July-August 2014; autumn, November-December 2014; winter, February-March 2015; and spring, May-June 2015. On each occasion 14–21 farms were visited, all available horses were inspected thoroughly, and all ticks were removed manually and stored in absolute ethanol until processing. During sampling, data were collected regarding the characteristics of each horse, the management of the farm (housing in stalls, paddocks or pasture), and the site of the body of the horse from which ticks were removed.

The sex, stage and species of each tick were determined according to its morphological characteristics [16–19].

### Environmental data

Geographical and environmental characteristics of each farm were analyzed using the geographical information system (GIS). Data obtained using remote sensing MODIS (moderate resolution imaging spectroradiometer satellite) technology comprised altitude, average salinity of soil, two vegetation indices (normalized difference vegetation index (NDVI) and enhanced vegetation index (EVI), and minimum, maximum and mean land surface temperature during the day (LSTD) and night (LSTN) [20]. Data obtained from the Israel meteorological service website (https://ims.data.gov.il/) for each sampling period comprised average rainfall, number of rainy days, and maximum daily rainfall, as well as minimum, maximum and mean ambient temperature, average moist temperature, average dew-point temperature, average humidity and average wind speed. Data of the soil type were obtained from the Israel government agency for mapping website (http://www.govmap.gov.il/).

### Statistical analysis

Data were analyzed in three ways: (i) Data for each individual tick were used to determine tick species and sex distribution, and its association with factors such as season and location on the horse. Associations between categorical parameters and each specific tick species were analyzed using *χ*^2^ or Fisher’s exact test, as appropriate. Relative abundance of each tick species was calculated as the seasonal percentage of the species collected throughout the year. (ii) Data pertaining to each farm, the average number of total ticks and specific tick species per horse (total number of ticks collected/number of examined horses), in each season were used to determine associations between tick infestation and farm location, geographical area and housing of the horses. Associations with climatic parameters were analyzed only for farms with pasture. Associations between categorical parameters and tick load were determined by Student’s t-test or analysis of variance (ANOVA), as appropriate. Correlations with climatic quantitative parameters were calculated using Spearman’s rho (ρ). (iii) Data presenting the tick load of each of the examined horses housed on pasture were used to analyze associations between tick burden and horse age, sex, breed and color. Associations between categorical parameters and tick load were determined by Student’s t-test or ANOVA, as appropriate, and odds ratios (OR) were calculated with their 95% confidence intervals (CI). All parameters that significantly correlated with total tick load were included in a multivariable forward stepwise logistic regression model, as well as a Generalized Estimating Equation (GEE), with the farm as a random variable. Statistical significance was set at *P* < 0.05. The analyses were performed using SPSS 22.0® and WinPepi 11.43® statistical software.

## Results

### Host study population

For the study, 396 horses were sampled on 24 farms: 218 horses on 14 farms during the summer; 293 horses on 20 farms during the autumn; 317 horses on 21 farms during the winter; and 268 horses on 19 farms during the spring. Of these, 156 horses were sampled for all four seasons, 93 horses were sampled for three seasons, 47 horses were sampled for two seasons, and 100 horses were sampled only once, making a total of 1096 samples (Additional file 1: Table S1).

Of the 396 horses, 191 (49%) were mares, 193 (48%) were geldings, and 12 (3%) were stallions. Most of the horses (251, 63%) were mixed-breed, while the rest were pure-bred, comprising 68 (17%) Quarter horses; 10 (2.5%) Paint horses; 13 (3%) Ponies; 10 (2.5%) Arabians; and several other breeds (fewer than 10 horses of each). Horses were of various colors: 54% were dark (124 chestnut, 73 bay, 9 black and 8 brown), 29% were light (61 gray, 32 palomino, 10 buckskin, 9 dun, 2 roan and one perlino), and 17% were spotted (36 pinto and 30 appaloosa).

### Spatio-temporal distribution of the tick species infesting horses

A total of 3267 ticks were collected during the study period. Ticks were found on 50% (12/24) of farms and on 25% (274/1096) of horses (Additional file 1: Table S1). The vast majority (99%) of ticks (3238) were collected from horses on pasture (*F*_(2,71)_ = 8.408, *P* < 0.001). Seven of the 24 examined farms kept horses on pasture and were located in three geographical regions: two in the Galilee, two on the Carmel mountain ridge (CMR) and three on the Golan Heights (GH) (Fig. 1). The majority of ticks (98%) were collected from five of these farms: 74% (2417 ticks) in the GH and 24% (782 ticks) in the CMR (*F*_(4,69)_*=* 8.598, *P* < 0.001). The number of infested horses at each farm is provided in Table 1.

**Table 1:** The prevalence of horses’ tick infestation on different farms during different seasons in Israel. The number of infested horses per the number on examined horses (%) are specified for each farm

Farm	Location	Summer (Jul-Aug 2014)	Autumn (Nov-Dec 2014)	Winter (Feb-Mar 2015)	Spring (May-Jun 2015)	Mean (%)
A^a,b^	33°13'25.8"N, 35°77'70.7"E	15/18 (83.3)	0/18 (0)	1/18 (5.5)	5/17 (29.4)	29.6
B	32°51'44.8"N, 34°94'99.9"E	0/9 (0)	0/9 (0)	0/7 (0)	1/9 (11.1)	2.9
C	32°52'95"N, 34°92'67.3"E	0/5 (0)	0/6 (0)	0/4 (0)	0/4 (0)	0
D^a,b^	32°73'16˝N, 35°00'63.1"E	24/25 (96)	0/41 (0)	23/38 (60.5)	36/36 (100)	59.3
E	32°95'91.2"N, 35°80'30.7"E	0/33 (0)	0/25 (0)	0/26 (0)	0/26 (0)	0
F	29°94'16.1"N, 35°06'46.9"E	0/16 (0)	0/15 (0)	0/15 (0)	0/14 (0)	0
G	32°90'48.1"N, 35°54'97"E	0/13 (0)	0/11 (0)	0/9 (0)	0/9 (0)	0
H	33°01'64.6"N, 35°26'38.8"E	2/11 (18.2)	1/10 (10)	0/7 (0)	0/9 (0)	8.1
I	32°92'95.9"N, 35°15'08"E	4/29 (13.8)	0/31 (0)	0/31 (0)	1/29 (3.4)	4.2
J	31°92'27.8"N, 34°82'11.9"E	11/9 (11.1)	–	–	–	11.1
K^a^	32°83'28.5"N, 35°78'35.7"E	3/14 (21.4)	1/11 (9.1)	0/11 (0)	7/9 (77.8)	24.4
L^a,c^	30°36'26.4"N, 35°15'51.4"E	7/9 (77.8)	7/9 (77.8)	5/8 (62.5)	7/7 (100)	78.8
M^a,c^	31°75'21"N, 35°15'01.6"E	18/18 (100)	28/28 (100)	19/28 (67.9)	28/28 (100)	91.2
N	32°55'68"N, 35°39'29.9"E	0/10 (10)	0/9 (0)	0/9 (0)	0/9 (0)	0
O	31°75'25.5"N, 35°46'62.9"E	–	0/17 (0)	0/17 (0)	0/17 (0)	0
P	32°82'17.9"N, 35°19'01.1"E	–	0/6 (0)	0/4 (0)	0/4 (0)	0
Q	32°67'58.5"N, 35°22'39.8"E	–	0/8 (0)	0/5 (0)	–	0
R^a^	31°88'32"N, 34°85'92.3"E	–	0/17 (0)	0/18 (0)	5/17 (29.4)	9.6
S	32°61'13.2"N, 34°99'14.4"E	–	0/6 (0)	0/7 (0)	0/8 (0)	0
T^a,c^	32°06'84.2"N, 34°82'47.8"E	–	9/10 (90)	–	10/10 (100)	95
U	32°07'66.8"N, 34°92'27.6"E	–	4/6 (66.7)	–	2/6 (33.3)	50
V	31°98'04.9"N, 34°92'76.9"E	–	–	0/38 (0)	–	0
W	31°30'40.9"N, 34°52'48.6"E	–	–	0/12 (0)	–	0
X	30°65'87.5"N, 35°23'96.5"E	–	–	0/5 (0)	–	0
Mean (%)		33.8	17.1	15.1	38	25

^a^Farms in which horses are kept on pasture

^b^Farms located in the CMR

^c^Farms located in the GH

**Fig. 2:**
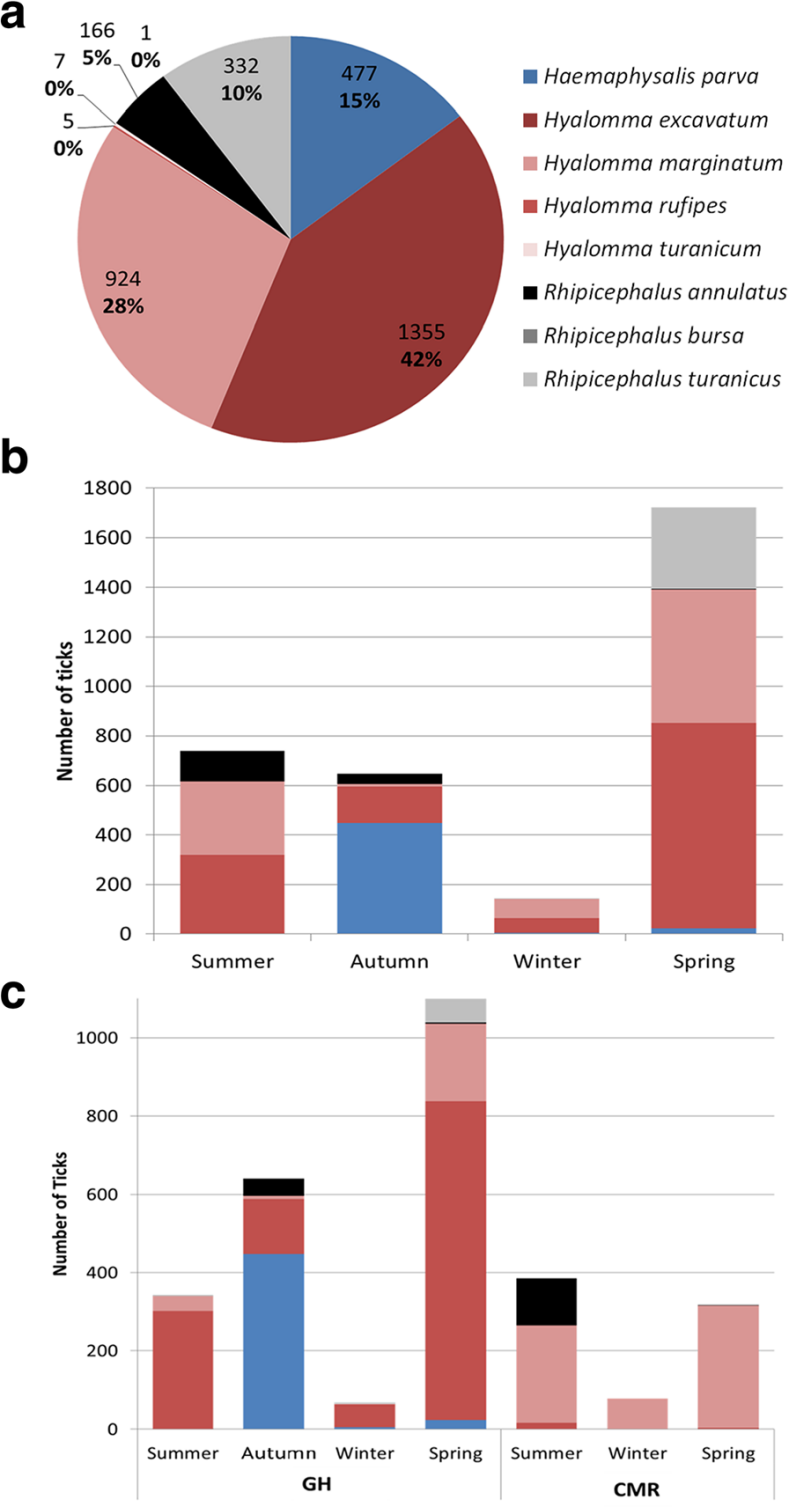
The distribution of tick species found on horses in Israel during a one-year period. **a** The number of ticks collected of each species and its percentage of the total ticks collected. **b** Seasonal distribution of equine tick infestation. **c** Tick species distribution in different seasons in the Golan Heights (GH) and in the Carmel Mountain Ridge (CMR)

Eight species of ticks were found to infest horses and the total number of ticks and species distribution varied among farms and seasons (Fig. 2). *Hyalomma* was the major tick genus, peaking in the spring and summer (*χ*^2^ *=* 826, *df* = 3, *P* < 0.001), with *Hy. excavatum* being the most prevalent species in the GH (*χ*^2^ *=* 665, *df* = 3, *P* < 0.001) and *Hy. marginatum* the most prevalent in the CMR (*P* < 0.001, two-sided). *Haemaphysalis parva* and *Rhipicephalus turanicus* ticks were found only in the GH, with *Ha. parva* peaking in the autumn (*P* < 0.001, two-sided) and *Rh. turanicus* peaking in the spring (*χ*^2^ *=* 311, *df* = 3, *P* < 0.001). *Rhipicephalus* (*Boophilus*) *annulatus* was found mainly on two farms (one in the CMR and the other in the GH), in which the horses were used for herding cattle (*χ*^2^ *=* 542, *df* = 1, *P* < 0.001). *Hyalomma turanicum* was rarely found, and was almost exclusive (6/7 ticks) on one farm located in the Great Rift Valley, near the Dead Sea.

**Fig. 3:**
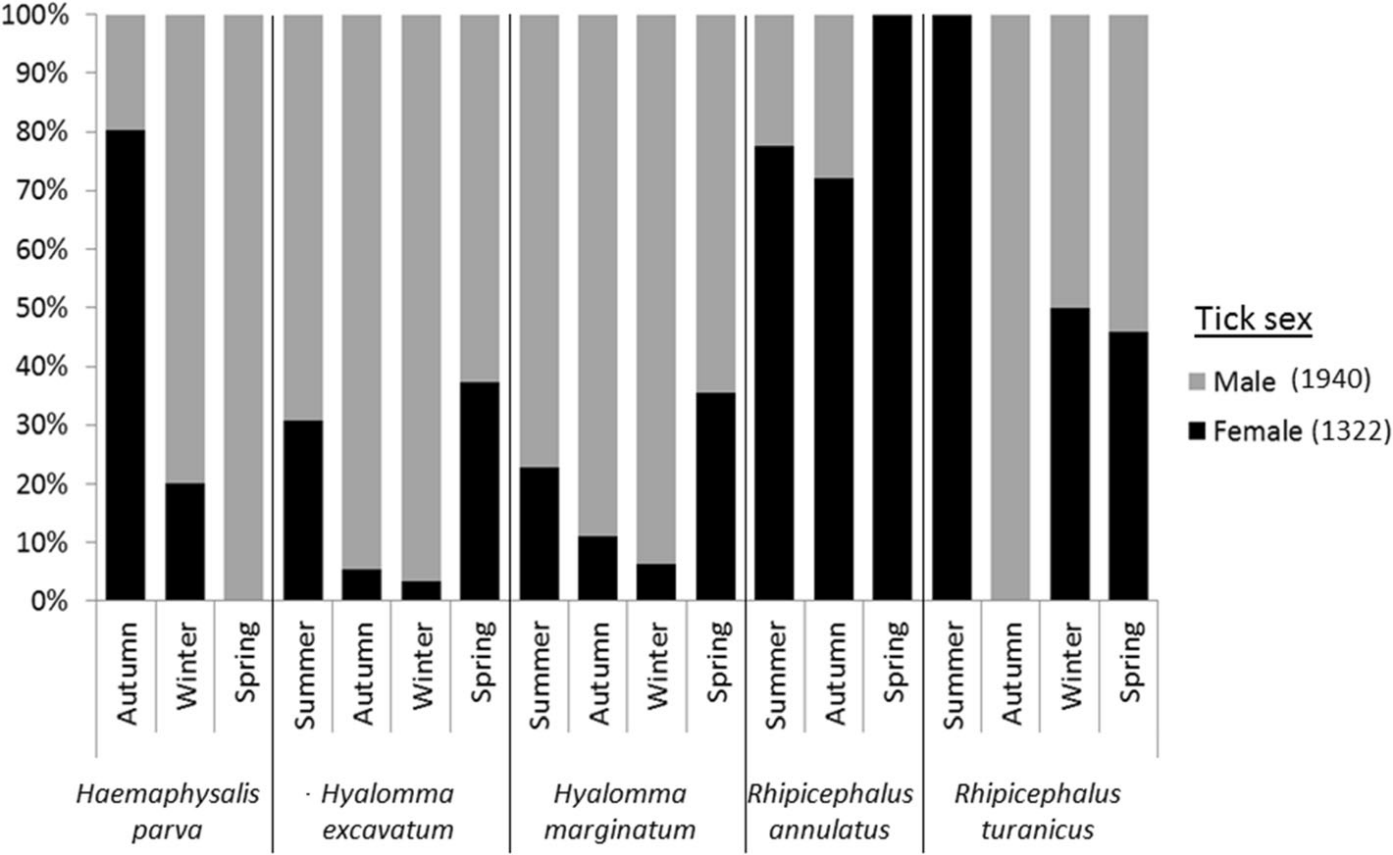
Percentage of male (gray) and female (black) ticks found on horses in Israel in different seasons

Almost all ticks found were adults (99.9%, 4 nymphs, 1 larva), 59% male (1940 ticks) and 41% female (1322) (Fig. 3). Female *Hyalomma* spp. and *Ha. parva* were more abundant than males in this tick’s peak season (spring: *χ*^2^ *=* 113, *df* = 3, *P* < 0.001; autumn: *χ*^2^ *=* 77, *df* = 2, *P* < 0.001, respectively).

### Association between host parameters and tick infestation

The host parameters significantly associated with tick infestation in the univariable analysis are summarized in Table 2. Mixed-breed horses displayed a higher tick burden than pure-bred horses, with similar association for all tick species. All tick species, except *Rh.* (*B*.) *annulatus*, infested mares more than male horses (stallions and geldings)*.* Spotted horses (pintos or appaloosas) had lower tick burdens than solid colored horses, but this burden was not different for *Hy. marginatum* and *Rh.* (*B.*) *annulatus* infestation. Horse age was not associated with tick infestation. Both breed (OR = 6.43, 95% CI: 2.05–10.82, *df* = 1, *P* = 0.004) and sex (OR = 5.34, 95% CI: 1.51–9.16, *df* = 1, *P* = 0.006) of the horse were found to be significant predictors of tick infestation in the multivariable GEE model.

**Table 2:** Host parameters significantly associated with tick infestation in univariable analysis. Tick were collected from 396 horses on 24 farms in Israel during one year

		Mean	SD	*t*-value	*df*	*P-*value
Breed	Mixed	7.41	12.16	12.389	446	<0.001
	Pure	0.15	0.36	–	–	–
Sex	Female	10.02	14.77	4.763	307	<0.001
	Male	4.55	8.24	–	–	–
Color	Spotted	3.52	7.44	-4.332	252	<0.001
	Solid	7.89	12.68	–	–	–

*Abbreviations: df* degrees of freedom, *SD* standard deviation

**Fig. 4:**
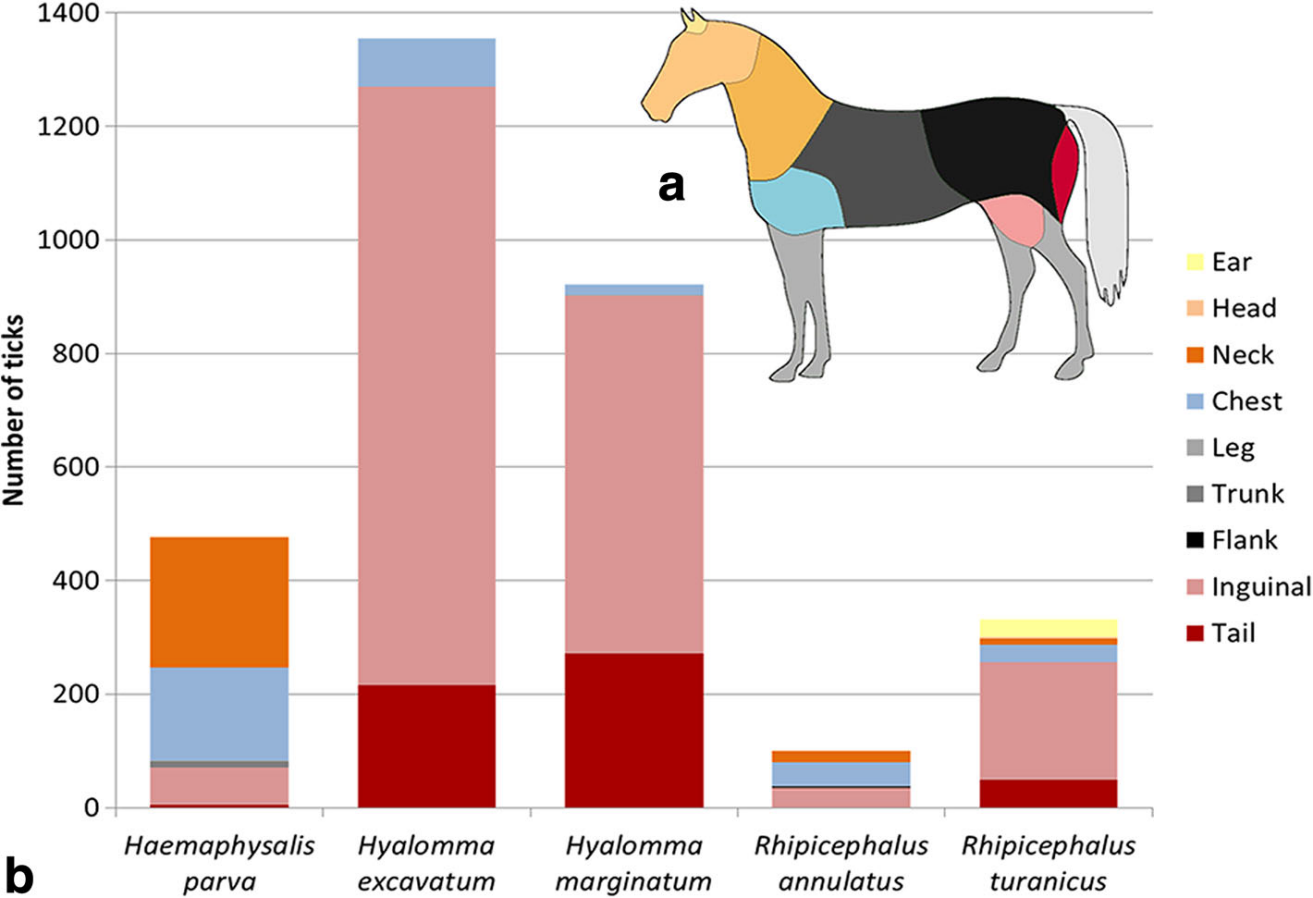
Tick species and the body site of the horse from which they were removed. **a** A schematic illustration of the different body sites of the horse, as referred to in this analysis. **b** The number of ticks of the different species collected from each body site of the horse

The distribution of ticks on different body parts of the horse varied among tick species (Fig. 4). *Hyalomma* ticks were found mainly on the rear parts of the horse: under the tail, in the inguinal area and hidden in the prepuce or between the udders (*χ*^2^ *=* 1312, *df* = 3, *P* < 0.001); *Haemaphysalis parva* ticks were found mostly on the proximal parts of the horse, including the neck, chest and trunk (*χ*^2^ *=* 1330, *df* = 8, *P* < 0.001); and *Rh. turanicus* ticks were found in both the ears and inguinal areas (*χ*^2^ *=* 300, *df* = 8, *P* < 0.001), but mainly on the rear (inguinal and under the tail) part of the horse (*χ*^2^ *=* 10, *df* = 3, *P* = 0.018).

### Environmental and climatic characteristics of the inspected farms

Farms were selected to represent the distribution of the equine population in Israel, with more farms in the north and center of the country and fewer in the south. Most of the farms were located in areas of Mediterranean climate (15 farms), and the rest in areas ranging from semi-arid to extreme arid (Fig. 1). Three of the five farms at which the majority of ticks were collected were located in the GH, and the remaining two in the CMR.

The GH is a volcanic plateau located between 300–1100 meters above sea level, with climate ranging from Mediterranean in the north to semi-arid in the south. The soil type is grumusol and basaltic protogrumusol. Most ticks were collected at one farm with 30 horses, located in the north of the GH, at an altitude of 980 m and with an average annual rainfall of 850 mm. The other two farms (10 horses each) were located further south, at an altitude of approximately 420 m and with an average rainfall of 625 mm.

The Carmel is a limestone mountain ridge located near the Mediterranean Sea. It peaks at 546 m, and features a Mediterranean climate and vegetation. The soil there is mainly terra-rossa and rendzina. One of the farms had 47 horses and was located 320 m above sea level, while the other had 18 horses and was located at an altitude of 119 m. The environmental characteristics of both areas are provided in Table 3.

**Table 3:** Environmental characteristics of the two Israeli Mediterranean climate regions examined in the current study: The Golan Heights (GH) and the Carmel mountain ridge (CMR)

	GH	CMR
Farms	3	2
Horses	49	65
Ticks	2417	782
Altitude (m)	420–980	119–320
Soil	Grumusol	Terra rosa
	Basaltic protogrumusol	Rendzina
Climate	Mediterranean to Semi-arid	Mediterranean
Annual rainfall (mm)	625–850	987
Average NVDI	0.04	0.06
Average EVI	0.024	0.03
LSTD (°C)	2–51	9–45
LSTN (°C)	-3–28	1–26
Average humidity (%)	66	69
Rainfall July-August	0	0
Ambient temperature July-August (°C)	14–37 (avg. 25)	19–34 (avg. 26)
Average rainfall November-December (mm)	90	86
Rainy days November-December	17–24	20–22
Ambient temperature November-December (°C)	-2–28.5 (avg. 13)	8–29 (avg. 17)
Average rainfall February-March (mm)	97	115
Rainy days February-March	20–28	20–22
Ambient temperature February-March (°C)	-5–29 (avg. 12)	3–29 ( avg. 15)
Average rainfall May-June (mm)	1.3	2
Rainy days May-June	1	4
Ambient temperature May-June (°C)	6–40 (avg. 21)	11–39 (avg. 22)

*Abbreviations*: *avg* average, *NDVI* normalized difference vegetation index, *EVI* enhanced vegetation index, *LSTD* land surface temperature in the day, *LSTN* land surface temperature in the night

### Associations between environmental parameters and tick infestation

Rainfall per season, number of rainy days, and maximum daily rain had a strong positive correlation (ρ > 0.86 and *P* < 0.001 for all associations), while maximum, minimum, and average ambient temperature, as well as moist temperature and dew point, positively correlated together (ρ > 0.61 and *P* < 0.007 for all, and ρ > 0.95 and *P* < 0.001 for all but the maximum ambient temperature) and negatively correlated with the rain parameters (ρ < -0.69 and *P* < 0.002).

The relative abundance of each tick species in the different seasons is provided in Table 4, and the association between tick infestation and environmental parameters is presented in Table 5. The average number of ticks per horse was moderately negatively correlated with the number of rainy days per season, mean NDVI (normalized difference vegetation index), and mean EVI (enhanced vegetation index), and showed a moderate positive correlation with maximum EVI and altitude. All these factors were also found to be statistically significant in the multivariable logistic regression model (all *P* < 0.001).

**Table 4:** Seasonal relative abundance of the different tick species infesting horses in Israel. Bold represents peak season of each species

	Summer	Autumn	Winter	Spring
*Haemaphysalis parva*	0	**0.94**	0.01	0.05
*Hyalomma excavatum*	**0.24**	0.11	0.04	**0.61**
*Hyalomma marginatum*	**0.32**	0.01	0.08	**0.58**
*Hyalomma rufipes*	**0.8**	0.2	0	0
*Hyalomma turanicum*	**0.71**	0.14	0	0.14
*Rhipicephalus annulatus*	**0.72**	0.26	0	0.02
*Rhipicephalus bursa*	0	0	0	**1**
*Rhipicephalus turanicus*	0.01	0.00	0.01	**0.98**
Total	0.23	0.20	0.04	0.53

**Table 5:** Spearman’s coefficient (ρ) and its significance (*P*) between environmental parameters and tick abundance on horses in Israel. Bold represents significant correlations

	Ticks		*Ha. parva*		*Hy. excavatum*		*Hy. marginatum*		*Rh. annulatus*		*Rh. turanicus*	
	ρ	*P*	ρ	*P*	ρ	*P*	ρ	*P*	ρ	*P*	ρ	*P*
Multiannual avg. rainfall	-0.01	0.98	0.15	0.55	0.08	0.74	0.29	0.24	**-0.51**	**0.03**	-0.03	0.92
Avg. NDVI	**-0.50**	**0.03**	-0.40	0.10	**-0.65**	**0.00**	0.25	0.33	-0.25	0.31	**-0.52**	**0.03**
Max. NDVI	0.21	0.40	0.11	0.65	0.04	0.89	0.05	0.85	0.19	0.45	-0.02	0.93
Min. NDVI	-0.42	0.08	-0.34	0.16	**-0.63**	**0.00**	0.16	0.51	-0.09	0.71	**-0.49**	**0.04**
Avg. EVI	**-0.50**	**0.03**	-0.40	0.10	**-0.65**	**0.00**	0.25	0.33	-0.25	0.31	**-0.52**	**0.03**
Max. EVI	**0.50**	**0.03**	0.40	0.10	**0.65**	**0.00**	-0.25	0.33	0.25	0.31	**0.52**	**0.03**
Min. EVI	-0.42	0.08	-0.34	0.16	**-0.63**	**0.00**	0.16	0.51	-0.09	0.71	**-0.49**	**0.04**
Max. LSTD	0.37	0.13	0.36	0.15	**0.56**	**0.02**	0.38	0.12	**-0.51**	**0.03**	0.27	0.28
Avg. LSTD	0.42	0.08	0.34	0.16	**0.63**	**0.00**	-0.16	0.51	0.09	0.71	**0.49**	**0.04**
Min. LSTD	-0.45	0.06	-0.41	0.09	**-0.58**	**0.01**	-0.30	0.23	0.35	0.16	-0.30	0.23
Max. LSTN	0.28	0.26	0.42	0.08	0.29	0.25	-0.15	0.55	0.05	0.84	0.23	0.36
Avg. LSTN	-0.19	0.46	-0.34	0.16	**-0.49**	**0.04**	-0.10	0.70	0.47	0.05	-0.34	0.17
Min. LSTN	-0.29	0.24	-0.34	0.16	**-0.60**	**0.01**	-0.30	0.22	**0.59**	**0.01**	-0.34	0.17
Altitude	**0.47**	**0.05**	0.45	0.06	**0.77**	**0.00**	0.28	0.26	**-0.49**	**0.04**	0.45	0.06
Soil salinity	-0.39	0.11	-0.41	0.09	**-0.72**	**0.00**	-0.13	0.61	0.40	0.10	**-0.48**	**0.05**
Avg. rainfall	-0.38	0.12	0.34	0.17	-0.20	0.42	**-0.52**	**0.03**	-0.29	0.24	-0.19	0.45
Rainy days	**-0.50**	**0.03**	0.35	0.15	-0.25	0.32	**-0.60**	**0.01**	-0.37	0.13	-0.21	0.40
Max. daily rain	-0.35	0.15	0.38	0.12	-0.16	0.53	**-0.53**	**0.02**	-0.24	0.33	-0.18	0.46
Max. temperature	0.34	0.17	**-0.50**	**0.04**	0.14	0.57	**0.49**	**0.04**	0.17	0.49	0.34	0.16
Min. temperature	0.07	0.79	**-0.59**	**0.01**	-0.21	0.41	0.44	0.07	0.28	0.27	-0.16	0.52
Avg. temperature	0.21	0.41	**-0.58**	**0.01**	-0.07	0.79	**0.50**	**0.04**	0.30	0.22	0.02	0.95
Moist temperature	0.17	0.51	**-0.58**	**0.01**	-0.14	0.58	**0.53**	**0.03**	0.31	0.20	-0.05	0.83
Dew point	0.15	0.56	**-0.61**	**0.01**	-0.14	0.57	**0.58**	**0.01**	0.26	0.30	-0.06	0.82
Avg. humidity	-0.19	0.45	0.15	0.54	-0.20	0.43	-0.02	0.94	0.14	0.58	-0.33	0.18
Avg. wind speed	0.37	0.19	0.05	0.86	**0.54**	**0.05**	0.29	0.32	-0.50	0.07	0.27	0.35

*Abbreviations*: *Min* minimum, *Max* maximum, *Avg* verage, *NDVI* normalized difference vegetation index, *EVI* enhanced vegetation index, *LSTD* land surface temperature in the day, *LSTN* land surface temperature in the night

Specifically, *Ha. parva* infestation moderately and negatively correlated with all ambient temperature parameters. *Hyalomma excavatum* infestation moderately and negatively correlated with mean and minimum NDVI and EVI, mean and minimum LSTN (land surface temperature in the night), minimum LSTD (land surface temperature in the day) and soil salinity and moderately and positively correlated with mean and maximum LSTD, altitude, and wind speed. *Hyalomma marginatum* infestation negatively correlated with all rain parameters, and positively correlated with all temperature parameters, except the minimum ambient temperature. *Rhipicephalus* (*B.*) *annulatus* demonstrated a moderate negative correlation with multi-annual average rainfall and maximum LSTD and altitude, and a moderate positive correlation with the minimum LSTN.

*Rhipicephalus turanicus* infestation had a moderate negative correlation with mean and minimum NDVI and EVI and soil salinity, and a moderate positive correlation with maximum EVI and mean LSTD.

## Discussion

The tick species infesting horses found in this survey represent species that are prevalent on livestock throughout the Mediterranean area [21–25], and differ from tick species found on horses elsewhere [26–28]. Almost all ticks found were adults, which suggests that the immature stages infest other host species. All the four nymphs and one larva that we found belonged to the species *Rh. annulatus*, which is a one-host tick, and thus all stages are expected to be found on the same host. The seasonal abundance of each species concurs with previous data on its peak season [24]. Tick sex ratio varied between seasons, with more females than males being found in the peak season of each species.

The vast majority of ticks were recovered from horses on pasture, which is the most prominent risk factor for tick infestation. Naturally, the chance of tick-host encounter increases on pasture environment, and most of the other studies of equine ectoparasites have focused on horses on pasture [26, 28]. Although the present study was designed to represent most of Israel’s geographical and climatic areas, the pasture factor obscured most of the other examined factors and, therefore, we focused only on the population of horses on pasture for most analyses. Specifically, we focused on two pasture areas in northern Israel under Mediterranean climate: the CMR and the GH regions, which are less than 100 km apart. These geographical regions differ in rock and soil composition, vegetation type, and many environmental parameters, and form different habitats for equine infesting ticks, as demonstrated in this study.

The predominant species in the CMR area was *Hy. marginatum* and the predominant species in the GH was *Hy. excavatum*. Although a similar number of horses was examined in both areas, the number of ticks found in the GH was 3-fold higher than that in the CMR, and the tick species found in the GH were more diverse. While all species found in the CMR were also found in the GH, *Rh. turanicus* and *Ha. parva* were found only in the GH (Fig. 2c). This difference in species diversity may reflect environmental or climatic preferences of different tick species or related to available hosts suitable for the tick immature stages.

*Hyalomma* is the most abundant tick genus infesting livestock in Mediterranean, semi-arid, and arid climates of Asia, Europe and Africa [24], and was the most abundant genus found on horses in this study (70%). *Hyalomma excavatum* was the most prevalent species, found in all examined parts of the country, but mostly in the GH. It is a two- or three-host tick species, whose adults feed mainly on ungulates, and it is the most prevalent tick species infesting cattle in Israel and Turkey [21, 24, 29, 30]. *Hyalomma marginatum* has been previously documented in Israel on cattle and horses [16], as well as on roe deer (*Capreolus capreolus*) in the CMR area [31]. This tick species is less tolerant to a dry climate [24], which may explain its presence in the more humid and arboreal environment typical of the CMR. The species is of growing significance since it is considered an important vector of CCHF, as well as of the equine piroplams, *Theileria* and *Babesia* species. Moreover, climate change have led to the spread of *Hy. marginatum* to new niches in Europe [2, 5, 6, 24]. Both *Hy. excavatum* and *Hy. marginatum* are established vectors of EP [32], which is endemic in Israel [7, 8].

*Hyalomma turanicum* and *Hy. rufipes* are closely related to *Hy. marginatum* [17, 24]. Only seven *Hy. turanicum* ticks were found in this study, six of which were on one farm located near the Dead Sea, in the Great Rift Valley area, which is characterized by a desert arid to extreme arid climate and low altitude (361 m below sea level). This species prefers steppe and desert climates [24], which may explain its restriction to this specific farm. *Hyalomma rufipes* vectors CCHF and several *Rickettsia* and *Babesia* species, and has been found on migratory birds in Israel and elsewhere [4, 24, 33]. Only five *Hy. rufipes* ticks were found in this survey in both the CMR and the GH. This number seems to be somewhat low considering that Israel is a major path for migratory birds between Africa, Asia, and Europe, and features a suitable climate for this species, although its adults are rarely found outside the Afrotropical region.

About 15% of the recovered ticks were of the genus *Rhipicephalus*. *Rhipicephalus turanicus* was the most prevalent species of this genus, and was found only in the GH and in the spring. It is found on a large variety of hosts and of climates [13, 16, 24, 29, 31, 34] and its restriction here to one geographical area was unexpected. *Rhipicephalus* (*B.*) *annulatus* was found only on horses working and grazing with cattle. It is a one-host tick with a clear preference for cattle, but can occasionally be found on horses [14, 24, 29]. It is also an important vector of several cattle hemoparasites [24, 35], but since it is mainly adapted to cattle, its role in vectoring equine pathogens is unknown.

Almost 15% of the ticks were *Ha. parva,* and were found only in the GH in the autumn and winter. This species was documented in Israel to infest mostly sheep and goats, but was also reported in horses and is found mostly in the cold season [16, 25, 29, 36].

Different tick species were found in specific places on the horse: *Hyalomma* spp. were collected mostly from the hindquarters; *Ha. parva* was found mostly on the neck and chest; while *Rhipicephalus* spp. were found on both the hindquarters and ears. These preferences have been previously documented [24, 25], and may reflect different tactics in protection against host defense, or simply different niches on the host of competing species.

Host parameters, such as sex, breed, and color that were found to be associated with tick infestation, mainly reflect the horse populations found on farms with access to pasture. Most pure-bred horses, as well as stallions, are kept in stalls and one of the farms with pasture mainly raises mixed-breed appaloosas.

Several environmental factors, including rain, temperature and vegetation, have been described to affect tick activity and life-cycle, with species-specific and also sub-population preferences [2, 5]. This may partly explain the different abundance of several *Hyalomma* species in the two niches studied. The climate niches in the CMR and the GH differ in many of the inspected parameters, including altitude, soil, the range of both ambient and land surface temperature and rainfall. While many of these parameters correlated with the abundance of different tick species, since data were collected only at four time points, many climatic factors correlated together; and since most ticks were collected in one season, the information we can deduct from these correlations is limited. A more elaborate study or a comparison with high-resolution data from other regions is needed in order to better identify the major climatic and geographical components that influence the preferences of the different species.

## Conclusions

This study is the first large-scale survey of ticks infesting horses in Israel, and provides an annual picture of horse-tick interactions. Pasture was found to be the main factor influencing exposure to ticks. Consequently, horse owners as well as veterinarians should consider a practice of parasite control, especially during the peak seasons of spring and summer. The CMR and the GH areas were shown to be distinct habitats in regard both to equine tick abundance and diversity. *Hyalomma* genus was the most prevalent tick observed to affect horses, and the two dominant species are known vectors of EP. *Haemaphysalis parva* and *Rh.* (*B.*) *annulatus* are not known as vectors of equine diseases, and their potential to transmit infectious agents between horses and other hosts should be further explored.

## Additional file

Additional file 1: **Table S1.** Ticks collected at each farm and season. For each farm the geographical coordinates are specified, along with the number of horses examined for each season, the number of ticks collected of each species, and the average number of ticks per horse. (XLSX 16 kb)

## Abbreviations

CCHF: Crimean Congo hemorrhagic fever
CMR: Carmel mountain ridge
EP: Equine piroplasmosis
EVI: Enhanced vegetation index
GH: Golan heights
*Ha*.: *Haemaphysalis*
*Hy*.: *Hyalomma*
LSTD: Land surface temperature during the day
LSTN: Land surface temperature during the night
NDVI: Normalized difference vegetation index
*Rh*. (*B*.): *Rhipicephalus* (*Boophilus*)
*Rh*.: *Rhipicephalus*
